# A Tumor Targeting Strategy of Phytoflavonoid Biochanin A for Efficient Fluorescence‐Guided Chemotherapy

**DOI:** 10.1002/smsc.202400111

**Published:** 2024-06-02

**Authors:** Yoonbin Park, Gayoung Jo, Hoon Hyun

**Affiliations:** ^1^ Department of Biomedical Sciences Chonnam National University Medical School Hwasun 58128 South Korea; ^2^ BioMedical Sciences Graduate Program (BMSGP) Chonnam National University Hwasun 58128 South Korea

**Keywords:** biochanin A, chemotherapy, hemicyanine, near‐infrared fluorescence imaging, tumor targeting

## Abstract

Cancer chemotherapy using natural phytochemicals, especially including isoflavone biochanin A (BCA), has attracted considerable attention because of the potent antitumor therapeutic effect and excellent biosafety. However, the preclinical application of BCA is still generally limited by its poor water solubility and low biological availability. To overcome these important limitations, a tumor targetable hemicyanine‐based near‐infrared (NIR) theranostic agent is rationally designed and prepared to improve the water solubility, tumor targetability, and antitumor activity of BCA. A key point to enhance the tumor targeting efficiency of BCA is the combination of a tumor‐targeted water‐soluble zwitterionic NIR fluorophore (ZW800‐Cl) and BCA to create the hemicyanine structure, named BCA‐ZW. Owing to the long‐wavelength emission (>750 nm) and large Stokes shift (72 nm) of BCA‐ZW, the in vivo performance of BCA‐ZW is effectively monitored. The molecularly engineered BCA‐ZW not only exhibits high targeting ability to HT‐29 xenograft tumors but also induces high levels of reactive oxygen species (ROS) generation in the tumor tissues. Therefore, the fluorescence‐guided chemotherapy by BCA‐ZW to the tumor‐bearing mouse model achieves the enhanced antitumor effect of BCA. This work provides a simple but effective strategy to design NIR fluorescent phytoflavonoids as potential therapeutic agents for further biomedical applications.

## Graphical abstract

A tumor targetable hemicyanine‐based near‐infrared (NIR) theranostic agent is rationally designed to improve the water solubility, tumor targetability, and antitumor activity of phytoflavonoid biochanin A (BCA). Herein, conjugation between BCA and a tumor‐targeted water‐soluble zwitterionic NIR fluorophore (BCA‐ZW) enables both real‐time tumor‐targeted fluorescence imaging as well as effective reactive oxygen species (ROS) generation for enhancing chemotherapeutic efficacy.

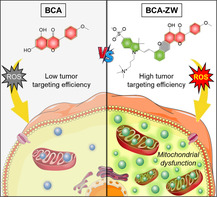

## 1 Introduction

Natural phytochemicals have been clinically used as first‐line anticancer drugs, including vincristine, etoposide, irinotecan, and paclitaxel derived from plants, because of their good biosafety and excellent chemotherapeutic efficacy in various cancer types.^[^ ^1^, ^2^, ^3^ ^]^ Although most of them still play an important role in cancer chemotherapy, novel natural products are required continuously to overcome the rapid development of chemotherapeutic drug resistance and their undesirable side effects.^[^ ^4^, ^5^ ^]^ Among the bioactive phytochemicals, flavonoids are highly effective in a wide range of diseases and considered as an alternative chemotherapeutic agent.^[^ ^6^, ^7^, ^8^ ^]^ Recently, biochanin A (BCA), a methylated derivative of isoflavone genistein, has attracted great attention because of its promising therapeutic potential, including antioxidant, antifibrotic, anti‐inflammatory, anti‐infective, and anticarcinogenic effects.^[^ ^9^, ^10^, ^11^, ^12^ ^]^ As a chemotherapeutic agent, the previous studies have reported in vitro anticancer efficacy of BCA on various cancer cell lines, including prostate, breast, lung, and pancreatic cancer cells, by the induction of apoptosis and inactivation of AKT/ERK signaling.^[^ ^13^, ^14^ ^]^ However, the clinical efficacy of BCA is limited by its low oral bioavailability and high body clearance, owing to poor aqueous solubility.^[^ ^15^, ^16^, ^17^ ^]^ To date, only a few studies have been conducted to increase the solubility of poorly water‐soluble BCA and further to enhance its bioavailability by encapsulating BCA into nanostructured lipid carriers, Pluronic F127/Plasdone S630‐mixed micelles, or polyvinylpyrrolidone‐based active solubilization technology (called NeoSol).^[^ ^18^, ^19^, ^20^ ^]^ Although these self‐assembled nanocarriers are the most common methods owing to the use of amphiphilic block copolymers, most of them still present ongoing issues such as manufacturing complexities, low tumor targetability, high nonspecific uptake, and biosafety.^[^ ^21^ ^]^ In this regard, a novel molecular design strategy is required to improve the in vivo performance of BCA.

Over the last decade, near‐infrared (NIR) emitting fluorophores are extensively used for noninvasive bioimaging applications because of their deep tissue penetration and high sensitivity and resolution with low background autofluorescence.^[^ ^22^, ^23^, ^24^ ^]^ To our knowledge, no study has yet reported on the effects of therapeutic NIR fluorophores combined with BCA through the formation of a hemicyanine scaffold. A molecular design strategy toward the tumor‐targeted imaging of BCA is to create the hemicyanine scaffold with a hydrophilic tumor‐targeted heptamethine cyanine fluorophore which not only increases the water solubility of BCA, but also enhances the tumor accumulation and therapeutic efficacy of BCA.

**Scheme 1:**
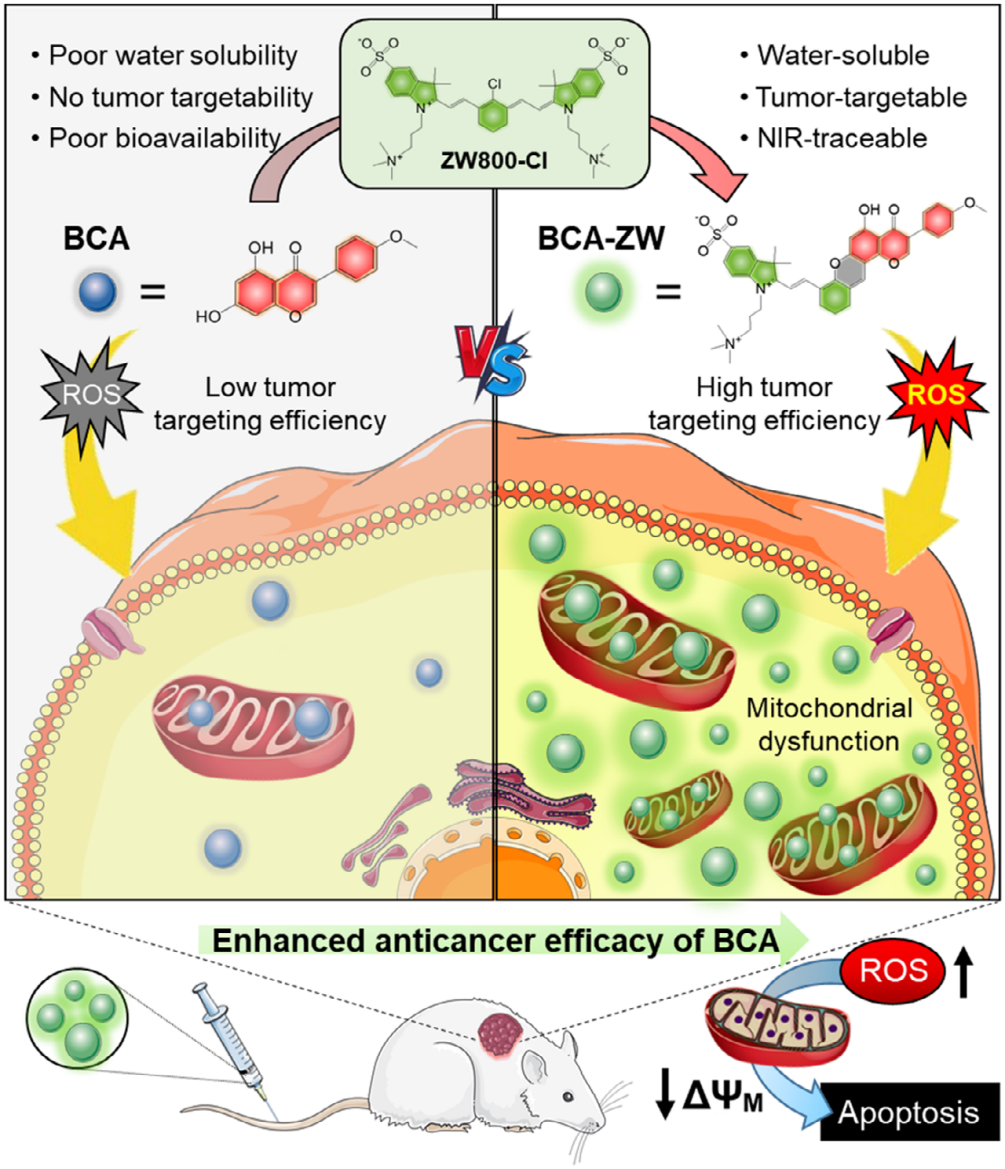
Schematic illustration of the action mechanism of BCA‐ZW. Non‐targeting BCA generates ROS at a low level. However, tumor targetable BCA‐ZW produces ROS at a higher level affecting the mitochondrial dysfunction, leading to a more efficient anticancer effect.

Based on the above strategy, we choose the zwitterionic heptamethine cyanine fluorophore, called ZW800‐Cl, to form the hemicyanine scaffold after combining with BCA and maintain its excellent water solubility and tumor‐specific uptake. Among commercially available heptamethine cyanine fluorophores, the zwitterionic side chain is considered as one of the neutral hydrophilic groups for the purpose of designing the water‐soluble NIR fluorescent BCA. In this study, we designed and synthesized a new type of NIR fluorescent BCA conjugate on the basis of the hemicyanine scaffold, named BCA‐ZW (**Scheme** **1**), with a zwitterionic hemicyanine as the hydrophilic and NIR fluorescent moieties, and the BCA group as the chemotherapeutic moiety, then compared the therapeutic efficacy between BCA‐ZW and BCA alone in an HT‐29 colorectal cancer xenograft model. The hemicyanine‐based BCA‐ZW conjugate not only exhibited efficient tumor targeting performance but also induced high levels of reactive oxygen species (ROS) production in the targeted tumor tissues. Therefore, this work provides a simple but effective strategy to design NIR fluorescent phytoflavonoids as potential therapeutic agents for improving anticancer efficacy.

## 2 Results and Discussion

### 2.1 Synthesis of ZW800‐Cl NIR Fluorophore and Its Hemicyanine Conjugates

**Figure 1:**
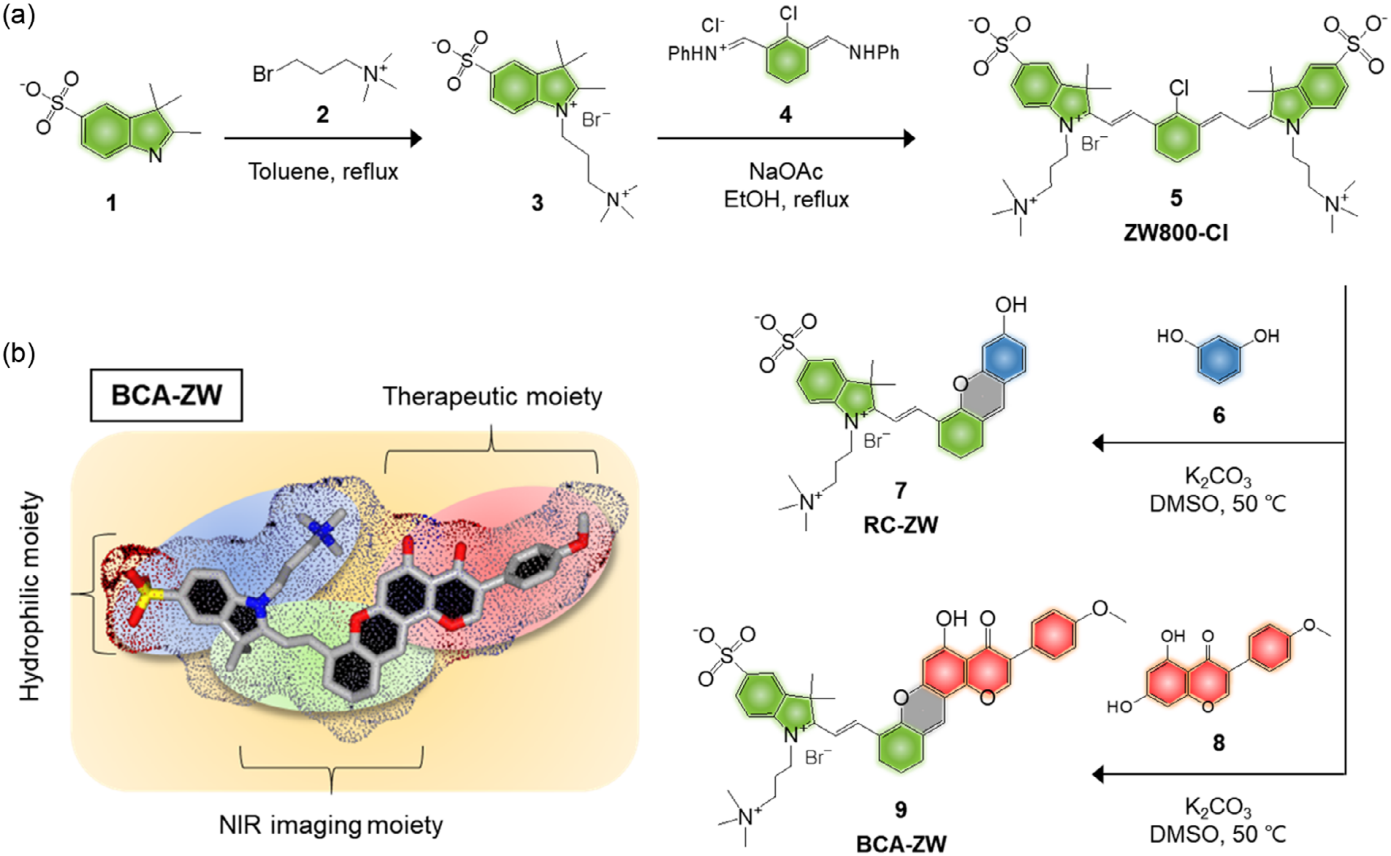
a) Synthetic scheme of ZW800‐Cl, RC‐ZW, and BCA‐ZW. b) The molecular design strategy of a hydrophilic tumor‐targeted therapeutic agent for improving water solubility, tumor targetability, and antitumor activity of BCA.

To synthesize the NIR fluorescent BCA conjugate, a tumor‐targeted water‐soluble zwitterionic NIR fluorophore, ZW800‐Cl used to form a hemicyanine scaffold, was prepared by the optimized synthesis and purification process reported previously.^[^ ^25^, ^26^ ^]^ The ZW800‐Cl was readily formed by the condensation reaction between the zwitterionic heterocyclic salt 3 and the Vilsmeier–Haack reagent 4 in the presence of anhydrous sodium acetate (**Figure** **1**a). Subsequently, the water‐soluble NIR fluorescent BCA‐ZW was synthesized between ZW800‐Cl and BCA 8. The chloro‐substituted ZW800‐Cl fluorophore is converted into a hemicyanine fluorophore under basic condition in the presence of potassium carbonate. Additionally, we synthesized the classical hemicyanine structure formed between ZW800‐Cl and resorcinol 6, named RC‐ZW, to clarify whether zwitterionic hemicyanine moiety exerts a significant influence on tumor targeting efficiency. Most importantly, the highly water‐soluble zwitterionic heptamethine cyanine fluorophore ZW800‐Cl was optimally selected among the commercially available other heptamethine cyanine dye analogues after performing in silico predictions of their distribution coefficient (log*D*) values with an aim to improve the water solubility of BCA (Figure S1, Supporting Information). The BCA‐ZW structure consists of three important moieties, which are the zwitterionic indole part for improving the water solubility and tumor targetability of BCA, the methine bridge for NIR fluorescence imaging, and the BCA moiety for the chemotherapeutic efficacy (Figure 1b). For the first time, we designed a water‐soluble NIR fluorescent BCA conjugate by using the zwitterionic NIR fluorophore for the tumor‐targeted imaging and chemotherapy. Moreover, the exact molecular weights of ZW800‐Cl, RC‐ZW, and BCA‐ZW were confirmed by mass spectrometry coupled with chromatography to validate their successful synthesis for the next in vitro and in vivo studies (Figure S2–S4, Supporting Information).

### 2.2 Optical Characteristics and Cytotoxicity of ZW800‐Cl NIR Fluorophore and Its Hemicyanine Conjugates

**Figure 2:**
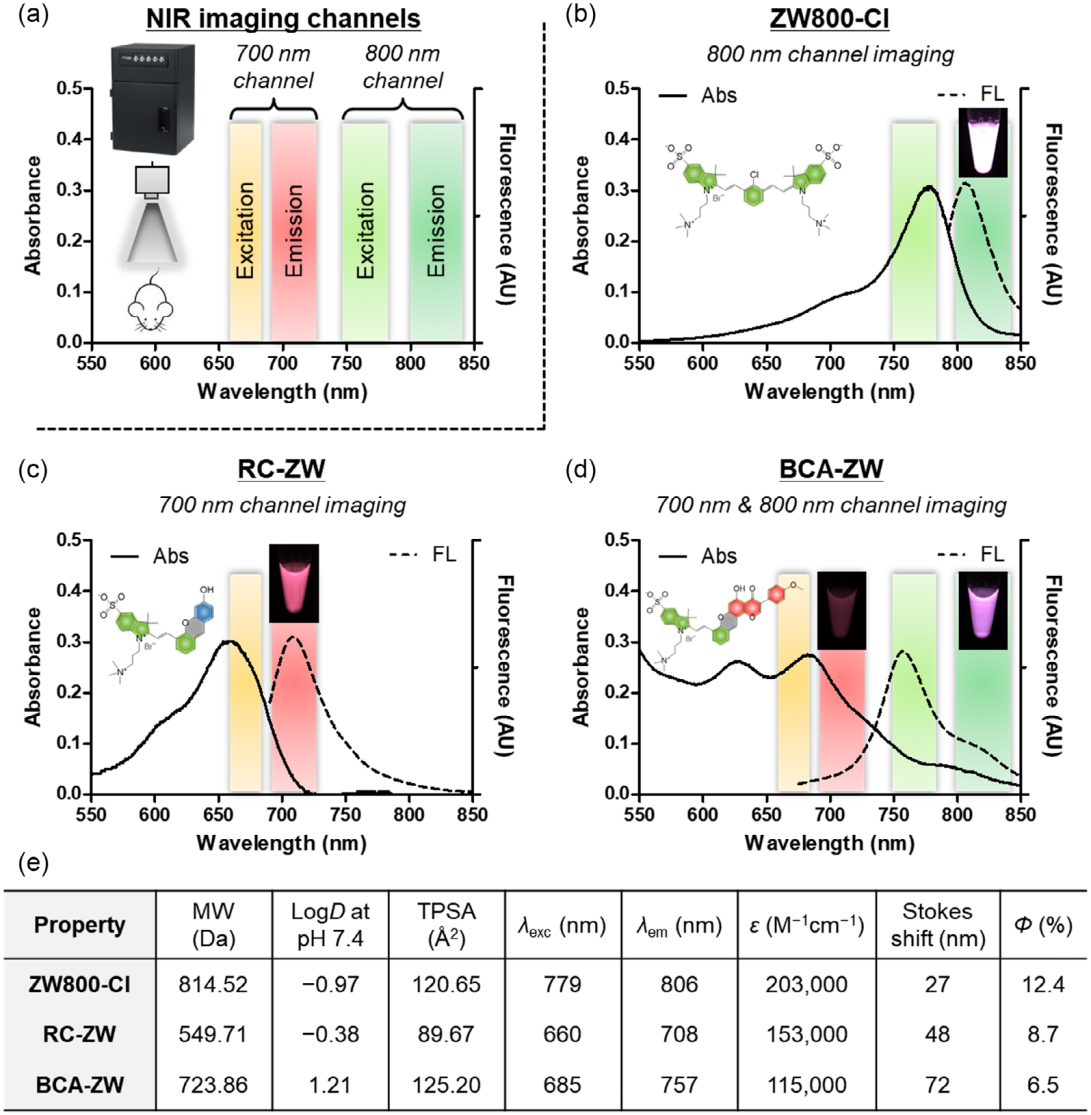
a) A dual‐channel NIR fluorescence imaging system. The red and green colors indicate excitation wavelength and detectable fluorescence emission window of independent imaging channels, respectively. Absorption and fluorescence emission spectra of b) ZW800‐Cl, c) RC‐ZW, and d) BCA‐ZW measured in PBS at pH 7.4. The inset shows the NIR fluorescence intensity of each sample under the independent imaging channel. e) Physicochemical and optical properties of ZW800‐Cl, RC‐ZW, and BCA‐ZW. In silico calculations of the distribution coefficient (log*D* at pH 7.4) and topological polar surface area (TPSA) were calculated using Marvin and JChem calculator plugins (ChemAxon).

After confirming the successful synthesis of ZW800‐Cl and its hemicyanine conjugates, we further explored their optical and physicochemical properties. It is important that the novel hemicyanine conjugate consisting of cyanine and BCA may extend the fluorescence emission wavelength compared to the conventional hemicyanine dyes, thereby providing very low background noise for in vivo fluorescence imaging. As shown in **Figure** **2**a, excitation and emission wavelength ranges for the in vivo NIR fluorescence imaging system are indicated by the red and green colors, which are excitation wavelength and detectable fluorescence emission window of independent imaging channels, respectively. As previously reported,^[^ ^26^, ^27^ ^]^ the highly water‐soluble zwitterionic heptamethine cyanine fluorophore ZW800‐Cl showed strong fluorescence emission intensity at 806 nm (Figure 2b). In addition, the classical hemicyanine structure of RC‐ZW has a maximum absorption peak at 660 nm and the maximum fluorescence emission at 708 nm when dissolved in phosphate‐buffered saline (PBS) at pH 7.4, respectively (Figure 2c). As expected, BCA‐ZW revealed that the absorption and fluorescence emission peaks at 685 and 757 nm shift to longer wavelengths with large Stokes shift (72 nm), compared to that of typical hemicyanine RC‐ZW (Figure 2d). The relative fluorescence quantum yield of BCA‐ZW was measured by using indocyanine green (ICG) dissolved in dimethyl sulfoxide (DMSO; *Φ* = 13%)^[^ ^28^, ^29^ ^]^ and determined to be 6.5% due to the low absorbance at 770 nm (Figure 2e). Although BCA‐ZW has a low quantum efficiency compared to that of ZW800‐Cl, the fluorescence intensity of BCA‐ZW under the in vivo fluorescence imaging system is sufficient to monitor the in vivo performance over time. Therefore, this result suggests that the novel hemicyanine conjugate BCA‐ZW has a significant benefit over the conventional hemicyanine dyes in terms of deep tissue imaging with low background autofluorescence. To investigate the aqueous stability of RC‐ZW and BCA‐ZW, the absorbance was measured after storing in PBS and FBS at ambient temperature for 7 days (Figure S5a, Supporting Information). Interestingly, RC‐ZW was more stable in both PBS and FBS, compared to that of BCA‐ZW. This indicates that BCA‐ZW may be biodegradable in the body. In addition, we monitored the fluorescence intensity of RC‐ZW and BCA‐ZW in PBS, FBS, and DMSO over the course of continuous illumination in the NIR imaging system to assess the photostability (Figure S5b, Supporting Information). The both hemicyanine conjugates showed no significant decrease in the fluorescence intensity tested in aqueous solutions, suggesting that the fluorescence brightness of RC‐ZW and BCA‐ZW in vitro and in vivo could be consistent for reliable imaging results.

Next, the potential cytotoxicity of BCA, RC‐ZW, and BCA‐ZW was examined using the 3‐(4,5‐dimethylthiazol‐2‐yl)‐2,5‐diphenyltetrazolium bromide (MTT) assay in NIH/3T3 and HT‐29 cells after incubation with various concentrations (5–50 μM) of each sample for 4 h (Figure S6, Supporting Information). Importantly, BCA‐ZW and BCA alone exhibited no significant cytotoxicity to the NIH/3T3 cells even at the high concentration of 50 μM, whereas the cell viability of HT‐29 cells markedly decreased with the increasing concentration of BCA‐ZW and BCA alone. Interestingly, RC‐ZW showed no significant cytotoxicity in both cell lines, while BCA‐ZW revealed lower cell viability of HT‐29 cells than that of BCA alone. This result demonstrates that BCA‐ZW has good biocompatibility on normal cells and the excellent chemotherapeutic effect on cancer cells.

### 2.3 Measurements of Mitochondria Membrane Potential and Intracellular ROS by JC‐1 and DCF‐DA Staining

**Figure 3:**
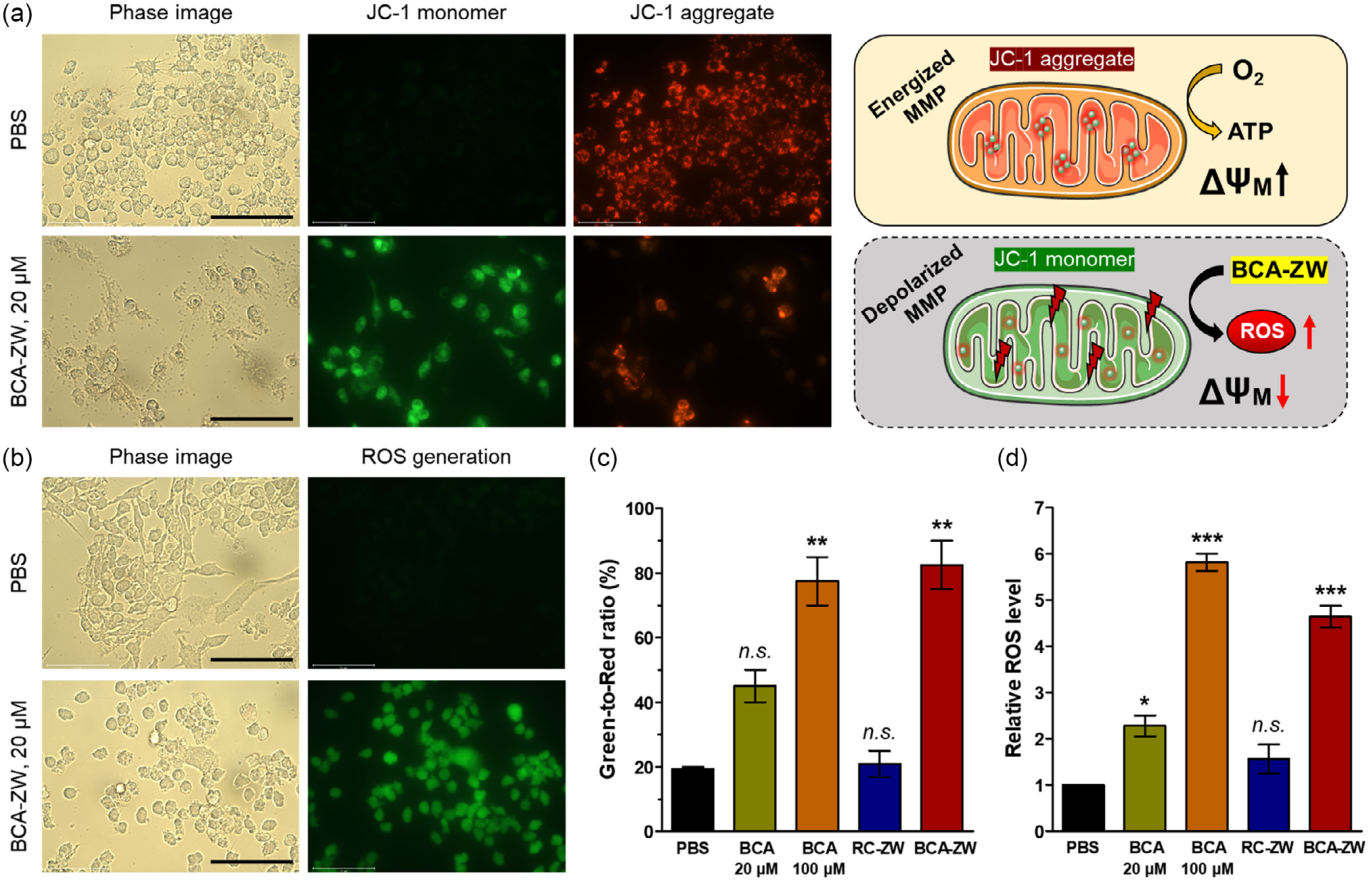
a) Measurement of mitochondria membrane potential by JC‐1 staining. b) Determination of cellular ROS by DCF‐DA assay. HT‐29 cells were incubated with PBS or 20 μM of BCA‐ZW for 24 h. Scale bars = 100 μm. Images are representative of *n* = 3 independent experiments. All fluorescence images have identical exposure times and normalization. c) Quantification of the green and red fluorescence intensity ratios in (a) and Figure S5. d) Quantification of relative fluorescence intensity in (b) and Figure S6. Data are expressed as mean ± S.D. (*p < 0.05, **p < 0.01, ***p < 0.001, *n* = 3).

To investigate the impact of BCA on mitochondrial function in HT‐29 cancer cells, a membrane‐permeable JC‐1 dye was used to evaluate the mitochondrial membrane potential (MMP) changes, because loss of MMP is a typical feature of mitochondrial damage. The JC‐1 dye is a cationic dye that displays MMP‐dependent fluorescence change between its aggregate state (red fluorescence with an emission of ≈590 nm; healthy cells with high MMP) and monomer state (green fluorescence with an emission of ≈529 nm; apoptotic cells with low MMP) to monitor the mitochondrial depolarization process. The fluorescence images of JC‐1 stained HT‐29 cells after different treatments are shown in **Figure** **3**a. Remarkably, the green fluorescence signals increased while the red fluorescence signals decreased in the treatment group of BCA‐ZW, compared to that in PBS group. This demonstrates that the BCA moiety of BCA‐ZW could effectively induce the loss of MMP, thereby contributing to mitochondrial dysfunction. In contrast, RC‐ZW without the BCA moiety does not depolarize the mitochondrial membrane, as proved by the weak green fluorescence intensity and strong red fluorescence intensity under the same conditions (Figure S7, Supporting Information). Additionally, the BCA alone groups showed concentration‐dependent changes in the red and green fluorescence ratios, indicating that the poorly water‐soluble BCA inefficiently induced the loss of MMP (Figure 3c; and S7, Supporting Information). Thus, these results clearly suggest that the water solubility of BCA improved by the zwitterionic moiety of BCA‐ZW which can promote the chemotherapeutic efficiency of BCA.

Previously, many studies have investigated the relationship between oxidative stress and cancer. Relatively high ROS levels can induce pro‐oncogenic signaling pathways to promote tumor formation, whereas excessive ROS levels can induce tumor cell death.^[^ ^30^ ^]^ In this regard, we further investigate whether excessive ROS generation of BCA‐ZW can directly lead to mitochondrial damage. The intracellular ROS production in cancer cells was typically measured by the nonfluorescent 2′,7′‐dichlorodihydrofluorescein diacetate (DCF‐DA) method. Importantly, the Figure 3b shows that BCA‐ZW induced a significant increase of ROS after 24 h of treatment, which was assessed by the oxidized form of DCF emitting green fluorescence. As expected, RC‐ZW exhibited a very weak DCF fluorescence intensity similar to that in PBS group, due to the absence of the BCA moiety (Figure 3d; and S8, Supporting Information). Additionally, BCA alone treated with a concentration of 20 μM displayed lower fluorescence intensity than that of BCA‐ZW in the HT‐29 cells, which is consistent with the result for JC‐1 in a concentration‐dependent manner. Therefore, these results reliably demonstrate that the zwitterionic moiety of BCA‐ZW exerts a marked impact on the intracellular ROS production of BCA.

### 2.4 Time‐Dependent In Vivo Biodistribution and Tumor Targeting of Hemicyanine Conjugates

**Figure 4:**
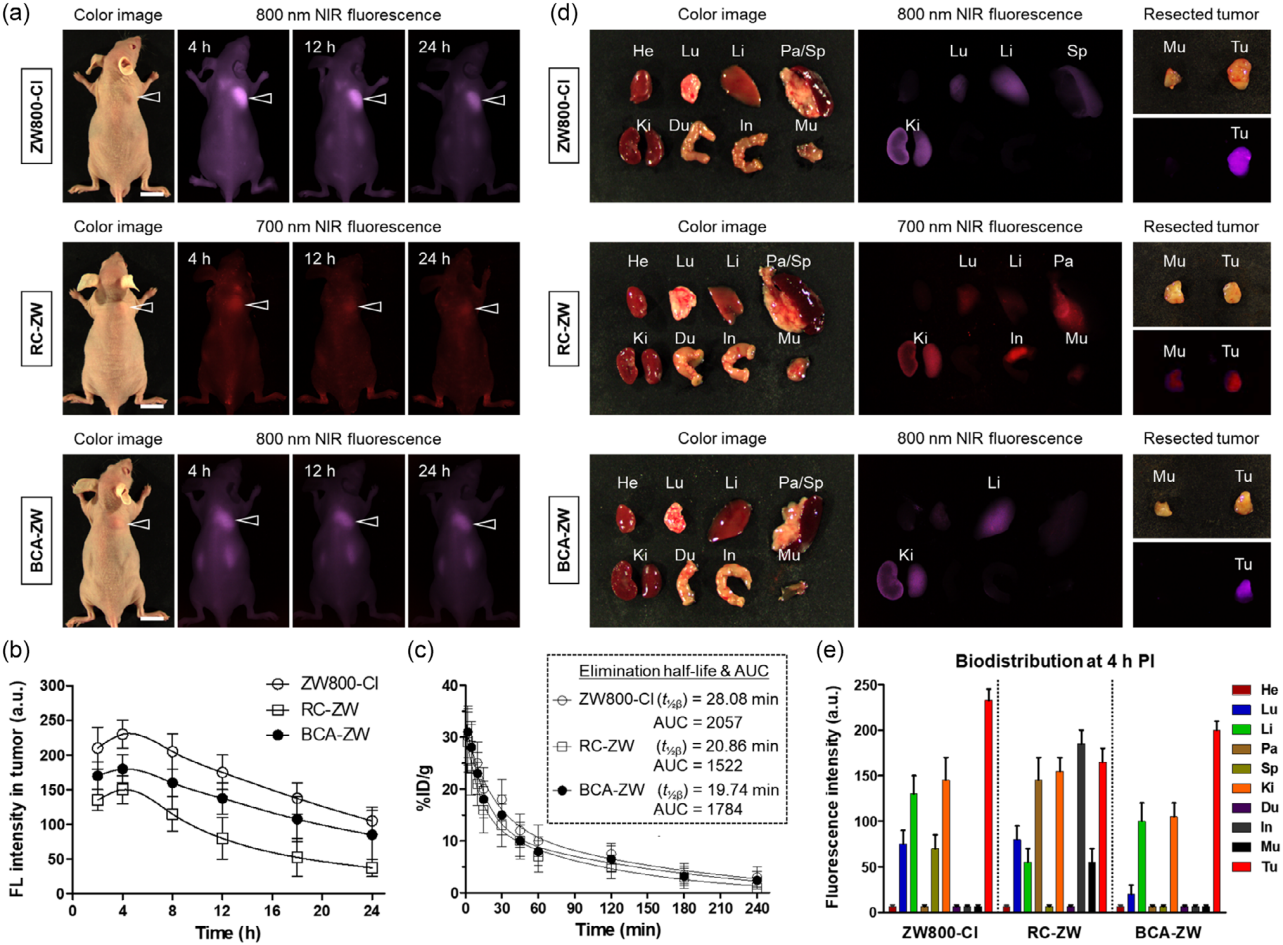
Time‐dependent in vivo tumor targeting of ZW800‐Cl, RC‐ZW, and BCA‐ZW. a) NIR fluorescence imaging for 24 h post‐injection of ZW800‐Cl, RC‐ZW, and BCA‐ZW. The tumor site is indicated by an arrowhead. Scale bars = 1 cm. b) Time‐dependent fluorescence intensity at tumor sites targeted by ZW800‐Cl, RC‐ZW, and BCA‐ZW. c) Pharmacokinetics of ZW800‐Cl, RC‐ZW, and BCA‐ZW. 25 nmol of each sample was injected intravenously into HT‐29 tumor‐bearing mice. Blood concentration (%ID/g) decay curve, elimination half‐life (*t*_1/2β_), and area under the curve (AUC) values are shown (*n* = 3, mean ± s.e.m.). d) In vivo biodistribution of ZW800‐Cl, RC‐ZW, and BCA‐ZW. Resected major organs and tumors imaged 4 h after injection of each sample. e) Quantitative fluorescence analysis of intraoperative dissected organs 4 h post‐injection of each sample. Abbreviations: Du, duodenum; He, heart; In, intestine; Ki, kidneys; Li, liver; Lu, lungs; Mu, muscle; Pa, pancreas; Sp, spleen; Tu, tumor; and PI, post‐injection. Images are representative of *n* = 3 independent experiments. All NIR fluorescence images have identical exposure times and normalization.

To evaluate the tumor targeting capability of BCA‐ZW, we investigated the accumulation of ZW800‐Cl, RC‐ZW, and BCA‐ZW at the tumor site by real‐time noninvasive NIR fluorescence imaging. After intravenous injection of the ZW800‐Cl and its hemicyanine conjugates, HT‐29 tumor‐bearing mice were subjected to whole‐body fluorescence imaging at different time points (**Figure** **4**a). The fluorescence signals at the tumor site in all three groups showed similar time‐dependent changes (Figure 4b). The highest fluorescence intensities at the tumor site were all observed at 4 h after injections of ZW800‐Cl, RC‐ZW, and BCA‐ZW, and then the tumor fluorescence signals gradually decreased over the time. As expected, the tumor‐specific uptake of ZW800‐Cl is consistent with the previous study, which describes that the meso‐chloride on a rigid cyclohexenyl ring of ZW800‐Cl plays an important role in forming covalent albumin adducts.^[^ ^27^ ^]^ It is well known that the tumor accumulation of the heptamethine cyanine dyes were explained by the receptor‐mediated endocytosis of albumin.^[^ ^31^, ^32^ ^]^ Surprisingly, the hemicyanine conjugates, RC‐ZW and BCA‐ZW prepared from ZW800‐Cl, also revealed preferential accumulation and retention in the tumor tissues, which result is similar to that of ZW800‐Cl. Although the exact mechanism for this finding remains unclear, we assume that the zwitterionic hemicyanine moiety plays a crucial role in the tumor‐specific uptake of RC‐ZW and BCA‐ZW.

Owing to the long‐wavelength emission and large Stokes shift of BCA‐ZW, the in vivo performance of BCA‐ZW is effectively monitored under the 800 nm NIR channel to ensure a better signal‐to‐background ratio as compared to that of RC‐ZW. Additionally, the pharmacokinetic profiles of ZW800‐Cl, RC‐ZW, and BCA‐ZW were evaluated after intravenous administration (1 μmol kg^−1^). As shown in Figure 4c, the elimination half‐life (*t*_1/2β_) and area under the curve (AUC) values were estimated to be 28.08 min and 2057%ID/g min for ZW800‐Cl, 20.86 min and 1522%ID/g min for RC‐ZW, and 19.74 min and 1784%ID/g min for BCA‐ZW in tumor‐bearing mice, respectively. The BCA‐ZW displayed the shortest blood circulation time compared to that of ZW800‐Cl and RC‐ZW, indicating that BCA‐ZW will be excreted promptly from the body, which decreases nonspecific uptake and potential toxicity. As the time reached at 48 h post‐injection, the fluorescence intensity of BCA‐ZW at the tumor site decreased substantially, suggesting the optimal timing of the second injection for a continuous infusion of chemotherapy. These results demonstrate that BCA‐ZW can be used for tumor‐specific imaging and targeted chemotherapy. Moreover, the biodistribution and clearance of ZW800‐Cl, RC‐ZW, and BCA‐ZW in the major organs was examined by ex vivo fluorescence imaging of organs and tissues harvested at 4 h post‐injection (Figure 4d,e). ZW800‐Cl and RC‐ZW exhibited high nonspecific uptake in the lungs, pancreas, spleen, and other major organs and tissues, whereas BCA‐ZW showed fluorescence signals mostly in the liver and kidneys as the main routes of drug excretion from the body. Also, the long‐term in vivo clearance of BCA‐ZW was evaluated by ex vivo fluorescence imaging of major organs collected at 24 and 48 h post‐injection (Figure S9, Supporting Information). As expected, the most of BCA‐ZW was eliminated from the body within 24 h of administration. This indicates that the zwitterionic hemicyanine moiety of BCA‐ZW not only contributes to the tumor‐specific uptake of BCA but also improves the biodistribution and clearance of BCA to prevent nonspecific organs/tissues uptake.

### 2.5 In Vivo Antitumor Efficacy of Hemicyanine Conjugates

**Figure 5:**
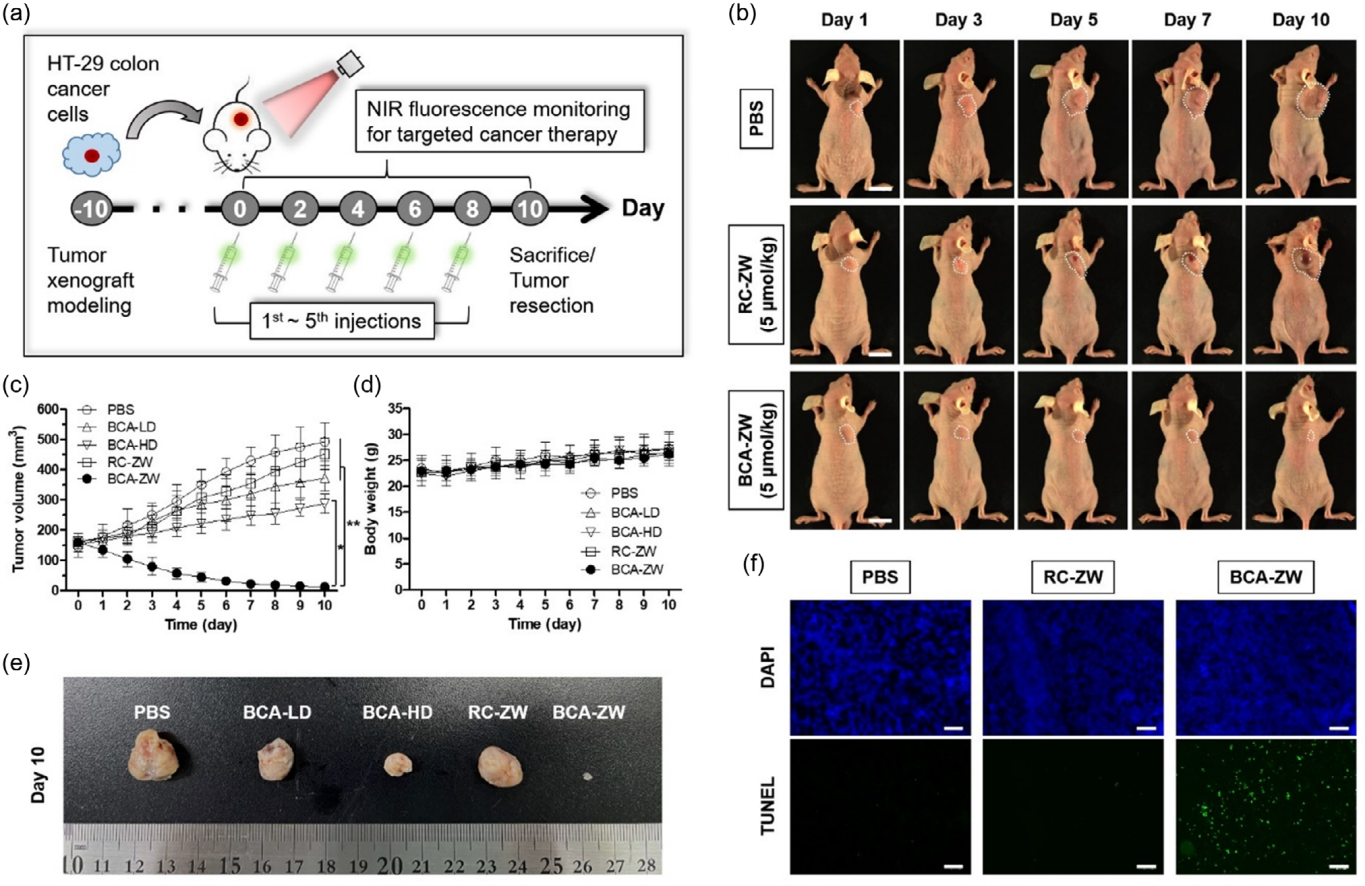
a) Scheme of the treatment timeline for in vivo antitumor efficacy of BCA, RC‐ZW, and BCA‐ZW. b) Representative photos of changes in tumor size in HT‐29 tumor‐bearing mice for 10 days after different treatments. The tumor sizes are indicated by white dotted lines. Scale bars = 1 cm. c) Tumor growth rates and d) body weights of each treatment group were monitored for 10 days. Data are expressed as mean ± S.D. (*p < 0.05, **p < 0.01, *n* = 3 for PBS, BCA, RC‐ZW, *n* = 5 for BCA‐ZW). e) The gross photo of tumors harvested from each treatment group at day 10. f) Apoptosis detection by TUNEL assay in tumor tissues harvested 7 days after different treatments. DAPI staining demonstrated the cell nucleus. Scale bars = 100 μm. Images are representative of *n* = 3 or 5 independent experiments. All fluorescence images have identical exposure times and normalization.

Results presented previously demonstrate that the increase of hydrophilicity in the hemicyanine moiety of BCA‐ZW leads to the enhanced tumor accumulation as well as the improved biodistribution and clearance of BCA‐ZW. Finally, we confirmed the potentiating effect of the zwitterionic hemicyanine moiety on the anticancer efficacy of BCA‐ZW in vivo using an HT‐29 xenografted nude mouse model. The treatment timeline of the tumor‐bearing mice in each group is depicted in **Figure** **5**a. At 10 days after inoculation of HT‐29 cancer cells, mice were treated with low‐dose BCA (BCA‐LD; 5 μmol kg^−1^), high‐dose BCA (BCA‐HD; 50 μmol kg^−1^), RC‐ZW (5 μmol kg^−1^), and BCA‐ZW (5 μmol kg^−1^) conjugates every other day by intravenous (for RC‐ZW and BCA‐ZW) or intraperitoneal (for BCA) injections for five times, based on their retention time in the tumor site. The three groups, in which mice were treated with PBS, RC‐ZW, and BCA‐LD, showed no significant differences in tumor growth (Figure 5b,c; and S10a, Supporting Information). As compared with those groups, tumor growth was partially delayed in mice treated with BCA‐HD. In contrast, tumor growth in the BCA‐ZW‐treated group significantly decreased for 10 days. Importantly, the high‐dose treatment of BCA‐HD (50 μmol kg^−1^) exhibited a weak inhibitory effect on tumor growth, whereas BCA‐ZW treated with the 10 times lower dose (5 μmol kg^−1^) revealed a strong inhibitory effect on solid tumor growth without recurrence for 10 days. This result may be attributed to high accumulation of the BCA‐ZW at the tumor site to enhance the antitumor effect of BCA. Additionally, the body weight of mice showed no noticeable change in all treatment groups (Figure 5d), indicating the superior biocompatibility of BCA and good biosafety of the hemicyanine conjugates. The H&E stained images of the major organs including heart, lung, liver, spleen, and kidney tissues also exhibited no appreciable signs of organ damage or inflammatory lesions in all treatment groups (Figure S11, Supporting Information). In addition, serum biochemical parameters, including alanine aminotransferase (ALT), aspartate aminotransferase (AST), blood urea nitrogen (BUN), and creatinine (CRE) were analyzed (Figure S12, Supporting Information). As expected, no significant differences were detected in the blood sample biochemistry between mice treated with PBS and BCA‐ZW. These results suggest that the BCA‐ZW can be used as a safe and effective chemotherapeutic agent. A photograph of resected tumors after various treatments at day 10 displayed clear differences in the tumor development among the five groups (Figure 5e). Furthermore, the tumor tissues resected from the different treatment groups were investigated with a terminal deoxynucleotide transferase‐mediated dUTP nick end labeling (TUNEL) assay, which is a well‐established technique to detect and quantify apoptotic cells within a cell population (Figure 5f; and S10b, Supporting Information). Strong green fluorescence emission was detected only in the BCA‐ZW group, and a very weak green fluorescence was observed in the BCA‐HD group, indicating cell apoptosis induced by BCA‐ZW.

In this study, we developed a tumor‐targetable NIR fluorescent BCA‐ZW conjugate for enhanced cancer chemotherapy of the BCA moiety by incorporating the zwitterionic hemicyanine moiety, which exhibited three activities. First, it helps to increase the water solubility of BCA, which can be leveraged to improve the bioavailability of poorly absorbed BCA with prolonged blood circulation. Secondly, it helps to improve the tumor targetability of BCA, which can reach the tumor specifically to achieve high tumor accumulation of BCA without nonspecific tissue/organ uptake, resulting in enhanced therapeutic efficacy of BCA. Third, upon excitation with light in the 750–780 nm range, it helps to achieve real‐time noninvasive monitoring of BCA via NIR fluorescence imaging and readily identify and quantify the biodistribution and tumor retention of BCA within the body. Overall, BCA‐ZW represents a novel all‐in‐one theranostic agent for the integration of tumor targeting, imaging, and therapy.

## 3 Conclusion

Until now, most other studies have been predominantly designed nanocarrier‐based systems for the encapsulation and targeted delivery of BCA to tumors using complicated methods. Unlike the nanosystem, we have successfully synthesized a newly designed NIR hemicyanine theranostic agent BCA‐ZW to improve the water solubility, tumor targetability, and antitumor activity of BCA. Moreover, we have demonstrated the effective antitumor activity of BCA‐ZW in vitro by enhancing intracellular ROS production and mitochondrial dysfunction in HT‐29 cells. More importantly, through regulating the zwitterionic moiety of hemicyanine, the molecularly engineered BCA‐ZW not only exhibited high targeting ability to HT‐29 xenograft tumors but also increased therapeutic efficacy of the BCA moiety, comparing to BCA alone. Therefore, this work provides a simple and effective strategy to design many other types of NIR fluorescent phytoflavonoids as potential therapeutic agents. For further study, the second strategy can be directed toward NIR laser‐activated photothermal cancer treatment using the BCA‐ZW for synergistic chemo‐photothermal therapy.

## 4 Experimental Section

4.1

4.1.1

##### Chemicals and Synthesis

All chemicals and solvents were of American Chemical Society grade or high‐performance liquid chromatography (HPLC) purity. Starting materials were purchased from Sigma‐Aldrich (St. Louis, MO, USA) and used as received without further purification. Final products were separated by preparative HPLC system (Waters, Milford, MA, USA) equipped with a PrepLC 150 mL fluid handling unit, a manual injector (Rheodyne 7725i) and a 2487 dual wavelength absorbance detector (Waters). ^1^H‐ and ^13^C‐NMR spectra were recorded on a Bruker Avance (400 MHz) spectrometer. The accurate mass of the purified product was analyzed by the Dionex UltiMate 3000 mass spectrometry system (Thermo Scientific, Waltham, MA, USA). See Supporting Information for detailed chemical syntheses and analyses.

##### Optical and Physicochemical Property Analyses

All optical measurements were performed in PBS at pH 7.4. The absorption spectra of ZW800‐Cl, RC‐ZW, and BCA‐ZW were recorded using a fiber optic FLAME absorbance and fluorescence (200–1025 nm) spectrometer (Ocean Optics, Dunedin, FL, USA). The molar extinction coefficient was calculated using the Beer–Lambert equation. To determine the fluorescence quantum yield (*Φ*), oxazine 725 in ethylene glycol (*Φ* = 19%)^[^ ^28^ ^]^ or ICG dissolved in dimethyl sulfoxide (DMSO; *Φ* = 13%)^[^ ^29^ ^]^ were used as calibration standards under the conditions of matched absorbance at 655 or 770 nm. The fluorescence emission spectra of ZW800‐Cl, RC‐ZW, and BCA‐ZW were analyzed using a SPARK 10M microplate reader (Tecan, Männedorf, Switzerland). In silico calculations of the distribution coefficient (log*D* at pH 7.4) and the topological polar surface area (TPSA) were performed using Marvin and JChem calculator plugins (ChemAxon, Budapest, Hungary).

##### In Vitro Live‐Cell Imaging

The human colorectal adenocarcinoma cell line (HT‐29) was obtained from the American Type Culture Collection (ATCC, Manassas, VA, USA). HT‐29 was cultured in Roswell Park Memorial Institute (RPMI) 1640 medium (Gibco BRL, Paisley, UK) containing fetal bovine serum, penicillin, streptomycin, and amphotericin B (Welgene, Daegu, South Korea) on a culture plate. The cultured cells were stored in a humidified incubator set to 5% CO_2_ at 37 °C. Fluorescence microscopic imaging was performed using a 4‐filter set of the Nikon Eclipse Ti‐U inverted microscope system (Nikon, Seoul, South Korea). Image acquisition and analysis were performed using the NIS‐Elements Basic Research software (Nikon). All fluorescence images had identical exposure time and normalization.

##### In Vitro Cytotoxicity Assay

When the HT‐29 cells reached a confluence of approximately 50%, cell toxicity and proliferation were evaluated using MTT (Sigma‐Aldrich) assay. HT‐29 cells were seeded onto 96‐well plates (1 × 10^4^ cells per well). To evaluate the cytotoxicity depending on the concentrations of each sample, the cancer cells were treated with BCA, RC‐ZW, and BCA‐ZW (5, 10, 20, and 50 μM) for 4 h and cultured for 24 h after treatment. At each time point, the incubation cell medium was replaced with 100 μL of fresh medium, and 10 μL of the MTT solution was directly added to each 100 μL well. Subsequently, the plates were then incubated for 4 h at 37 °C in a humidified 5% CO_2_ incubator. Finally, the plates were placed in a microplate reader (SPARK 10M, Tecan) to measure the absorption intensity at 570 nm. Cell viability was calculated using the following formula: cell viability (%) = (*A*_sample_–*A*_blank_)/(*A*_control_–*A*_blank_) × 100, where *A* is the average absorbance.

##### Measurement of Mitochondrial Membrane Potential

HT‐29 cells were treated with the 20 or 100 μM concentrations of BCA, RC‐ZW, and BCA‐ZW for 24 h. After the treatment, the cells were washed with PBS and incubated with a 10 μg mL^−1^ concentration of 5,5′,6,6′‐tetrachloro‐1,1′,3,3′‐tetraethylbenzimidazolylcarbocyanine iodide (JC‐1, Thermo Fisher Scientific) for 30 min at 37 °C. JC‐1 dye was used as an indicator of mitochondrial membrane potential in HT‐29 cells. The cells were repeatedly washed with PBS and then observed under the fluorescence microscope (Nikon).

##### Cellular ROS Assay

HT‐29 cells were incubated with the 20 or 100 μM concentrations of BCA, RC‐ZW, and BCA‐ZW for 24 h. Subsequently, the cells were washed with PBS and treated with a 100 μM concentration of 2′,7′‐dichlorodihydrofluorescein diacetate (DCF‐DA, Thermo Fisher Scientific) for 30 min at 37 °C. Cellular ROS production is commonly estimated by nonfluorescent DCF‐DA, which permeates the cell membrane and reacts with reactive oxygen to give a DCF form emitting green fluorescence. After the treatment of DCF‐DA, the cells were repeatedly washed with PBS and then observed under the fluorescence microscope (Nikon).

##### HT‐29 Xenograft Mouse Model

Animal experiments were carried out in accordance with the guidelines approved by the Chonnam National University Animal Research Committee (CNU IACUC‐H‐2023‐57). Male athymic nude mice (6 weeks old and ≈25 g, *n* = 3 independent experiments) were received from OrientBio (Gwangju, South Korea) to prepare human tumor xenograft models. The cultured HT‐29 cancer cells were dispersed in PBS before inoculation subcutaneously into the mouse upper right flank area (1 × 10^6^ cells per mouse). Finally, mice bearing subcutaneous tumors sized with an average diameter of 1 cm were subjected to intravenous (for ZW800‐Cl, RC‐ZW, and BCA‐ZW) or intraperitoneal (for BCA) injections of each sample at 8–10 days post‐inoculation. The tumor‐bearing mice were anesthetized at specific time points for real‐time whole‐body imaging.

##### In Vivo Biodistribution and Tumor Imaging

In vivo NIR fluorescence imaging was performed using a FOBI imaging system (NeoScience, Deajeon, South Korea). At 4 h post‐injection, the mice were sacrificed and their major organs (heart, lungs, liver, pancreas, spleen, kidneys, duodenum, and intestine) were collected and imaged to evaluate the time‐dependent biodistribution of each sample. Fluorescence intensities accumulated in tumor and organs were analyzed using ImageJ version 1.45q (National Institutes of Health, Bethesda, MD, USA). All NIR fluorescence images were normalized identically for all conditions.

##### In Vivo Therapeutic Efficacy

Samples were intravenously (for RC‐ZW and BCA‐ZW) or intraperitoneally (for BCA) injected into the HT‐29 tumor‐bearing mice (three mice per treatment group for PBS, BCA, and RC‐ZW; five mice per treatment group for BCA‐ZW), and mice were monitored daily for 10 days. Tumors were excised from the treated mice 7 day after injection for subsequent histological analysis. To assess the in vivo antitumor effect, the macroscopic tumor growth of each treatment group was observed for 10 days. The tumor volume (V) was measured by the following formula: *V* = 0.5 × longest diameter × (shortest diameter)^2^.

##### TUNEL Assay

Tumor tissues were collected from each of the five treatment groups to confirm an observation of apoptotic cell death. They were fixed in 4% paraformaldehyde at −20 °C for 30 min and cryosectioned (10 μm in thickness per slide). Then, the samples were washed with PBS and incubated with a terminal deoxynucleotide transferase‐mediated dUTP nick end labeling (TUNEL) reagent containing terminal deoxynucleotidyl transferase and fluorescent isothiocyanate dUTP using a DeadEnd Fluorometric TUNEL System (Promega, Madison, WI, USA). After incubation, they were stained with 4′,6‐diamidino‐2‐phenylindole (DAPI; 1 μg mL^−1^) for 30 min to investigate the cell nucleus by UV light microscopic observations (blue). Fluorescence imaging was performed on a Nikon Eclipse Ti‐U inverted microscope system. Image acquisition and analysis were performed using NIS‐Elements Basic Research software (Nikon).

##### Assessment of Safety In Vivo

Resected major organs (heart, lung, live, spleen, and kidney) harvested from each treatment group at day 10 were preserved for hematoxylin and eosin (H&E) staining and microscopic assessment. Samples were fixed in 4% paraformaldehyde and flash frozen in optimal cutting temperature (OCT) compound using liquid nitrogen. Frozen samples were cryosectioned (10 μm in thickness per slide), stained with H&E, and observed by microscopy. Histological imaging was performed on a Nikon Eclipse Ti‐U inverted microscope system. Image acquisition and analysis was performed using NIS‐Elements Basic Research software (Nikon). To evaluate the potential toxicity of BCA‐ZW in vivo, blood samples were collected and centrifuged at 4500 rpm at 4 °C. Subsequently, the supernatant was stored at −80 °C for analysis of blood biochemical parameters.

##### Statistical Analysis

Statistical analysis was performed using a one‐way analysis of variance (ANOVA) followed by Tukey's multiple comparisons test. Differences were considered to be statistically significant at a level of *p* < 0.05. Results were presented as the mean ± S.D., and the curve fitting was performed using Prism version 8.0.2 (GraphPad, San Diego, CA, USA).

## Conflict of Interest

The authors declare no conflict of interest.

## Author Contributions

Conceptualization, H.H.; methodology, H.H.; validation, Y.P. and G.J.; formal analysis, Y.P. and G.J. investigation, Y.P. and G.J.; data curation, Y.P. and G.J.; writing – original draft preparation, Y.P., and H.H.; writing – review & editing, Y.P., and H.H.; visualization, Y.P. and H.H.; supervision, H.H.; project administration, H.H.; funding acquisition, H.H. All authors have read and agreed to the published version of the manuscript.

## Supporting information

Supplementary Material

## Data Availability

The data that support the findings of this study are available in the supplementary material of this article.
