# Supplementary material for: A Tumor Targeting Strategy of Phytoflavonoid Biochanin A for Efficient Fluorescence‐Guided Chemotherapy
Source: Small Sci. 2024 Jun 2;4(8):2400111. doi: 10.1002/smsc.202400111 (PMC11935084; doi:10.1002/smsc.202400111)
Supplement: Supplementary file 1 — Supplementary Material [file SMSC-4-2400111-s001.pdf]

Supporting Information

A Tumor Targeting Strategy of Phytoflavonoid Biochanin A for Efficient Fluorescence-Guided Chemotherapy

*Yoonbin Park, Gayoung Jo, and Hoon Hyun**

Yoonbin Park, Gayoung Jo, Hoon Hyun
Department of Biomedical Sciences, Chonnam National University Medical School, Hwasun 58128, South Korea
Yoonbin Park, Hoon Hyun
BioMedical Sciences Graduate Program (BMSGP), Chonnam National University, Hwasun 58128, South Korea

Contents:

Supplementary Methods

Figure S1. Rational design strategy of hemicyanine-based near-infrared (NIR) fluorophores for conjugation with biochanin A (BCA).

Figure S2. Mass spectrum of ZW800-Cl.

Figure S3. Mass spectrum of RC-ZW.

Figure S4. Mass spectrum of BCA-ZW.

Figure S5. Stability and photostability of RC-ZW and BCA-ZW.

Figure S6. Cell viability assay of BCA, RC-ZW, and BCA-ZW.

Figure S7. Measurement of mitochondria membrane potential by JC-1 staining.

Figure S8. Determination of cellular reactive oxygen species (ROS) by DCF-DA assay.

Figure S9. In vivo biodistribution of BCA-ZW at 24 h and 48 h post-injection.

Figure S10. In vivo antitumor efficacy of low-dose (LD; 5 μmol/kg) and high-dose (HD; 50 μmol/kg) BCA.

Figure S11. H&E stained images of major organs.

Figure S12. Blood biochemical analysis after intravenous injections of PBS and BCA-ZW.

Supplementary Methods

As shown in **Figure 1a**, the synthesis of ZW800-Cl, RC-ZW, and BCA-ZW was accomplished using the published synthetic methods.[S1-S3] The ZW800-Cl and its hemicyanine-based conjugates were synthesized as follows:

*2,3,3-Trimethyl-1-[3-(trimethylammonio)propyl]-3H-indolium-5-sulfonate (**3**).* A mixture of 2,3,3-Trimethyl-3*H*-indole-5-sulfonate **1** (23 g, 83 mmol) and (3-bromopropyl)trimethyl ammonium (**2**; 25 g, 96 mmol) in toluene (350 mL) was heated at 70°C for 48 h under a nitrogen atmosphere. The mixture was cooled to room temperature and the solvent was decanted. Methanol (100 mL) was added into the crude mixture and stirred for 30 min. The crude mixture was filtered, collected, and redissolved in a 2:1 (v/v) mixture of water (100 mL) and methanol (50 mL). The mixture solution was slowly added into acetonitrile (1.6 L) using a dropping funnel. The precipitate was filtered and collected as a pink solid (16 g, 36%); ^{1}H NMR (400 MHz, DMSO-d_6): δ 1.56 (s, 6*H*), 2.51 (s, 3*H*), 3.07 (m, 2*H*), 3.12 (s, 9*H*), 3.62 (t, J = 7.2 Hz, 2*H*), 4.50 (t, J = 7.2 Hz, 2*H*), 7.71 (d, J = 8.0 Hz, 1*H*), 7.79 (d, J = 8.0 Hz, 1*H*), 8.01 (s, 1*H*). ^{13}C NMR (400 MHz, DMSO-d_6): δ 15.0, 21.6, 22.3, 45.2, 53.1, 55.0, 62.4, 115.4, 121.2, 126.8, 141.3, 142.01, 149.9, 199.1.

*2-((E)-2-((E)-2-chloro-3-((E)-2-(3,3-dimethyl-5-sulfonato-1-(3-(trimethylammonio)propyl) indolin-2-ylidene)ethylidene)cyclohex-1-en-1-yl)vinyl)-3,3-dimethyl-1-(3-(trimethyl-ammonio) propyl)-3H-indol-1-ium-5-sulfonate bromide (**5**; ZW800-Cl).* A mixture of bromide salt **3** (15 g, 27.9 mmol), Vilsmeier-Haack reagent **4** (5 g, 13.9 mmol), and anhydrous sodium acetate (3.42 g, 41.7 mmol) in absolute ethanol (200 mL) was heated under reflux for 6 h under a nitrogen atmosphere. The reaction mixture was cooled to room temperature, and then filtered, washed with ethanol and methanol, and collected as a brownish-green solid (10.2 g, 90%); ^{1}H NMR (400 MHz, DMSO-d_6): δ 1.72 (s, 12*H*), 1.88 (m, 2*H*), 2.18 (m, 4*H*), 2.76 (m, 4*H*), 3.08 (s, 18*H*), 3.49 (m, 4*H*), 4.18 (m, 4*H*), 6.36 (d, J = 14 Hz, 2*H*), 7.45 (d, J = 8.0 Hz, 2*H*), 7.70 (d, J = 8.0 Hz, 2*H*), 7.85 (s, 2*H*), 8.31 (d, J = 14 Hz, 2*H*). ^{13}C NMR (600 MHz, D_2O + DMSO-d_6): δ 17.74, 20.72, 23.58, 30.46, 55.10, 55.68, 59.45, 65.58, 68.22, 104.97, 113.62, 122.92, 129.66, 131.19, 143.94, 145.55, 145.67, 147.38, 176.09.

*Synthesis of RC-ZW (**7**) and BCA-ZW (**9**).* A mixture of resorcinol (0.22 g, 2.0 mmol) for RC-ZW or biochanin A for BCA-ZW (0.56 g, 2.0 mmol), and potassium carbonate (0.28 g, 2.0 mmol) in dimethyl sulfoxide (DMSO; 20 ml) was stirred at 50°C for 30 min. Then ZW800-Cl

(0.81 g, 1.0 mmol) was dissolved in DMSO (10 ml) and added to the above mixture. The reaction mixture was stirred at 50°C for additional 3 h. The crude products were purified by a preparative high-performance liquid chromatography system, giving RC-ZW (0.35 g, 64%) and BCA-ZW (0.42 g, 58%).

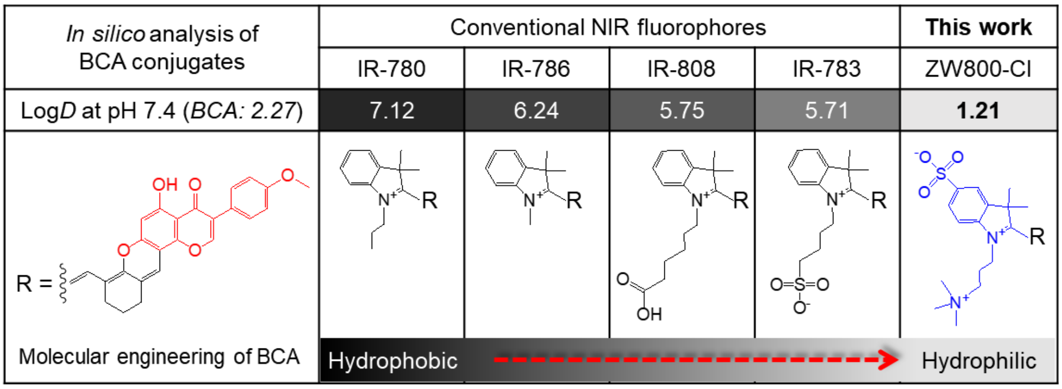

Figure S1. Rational design strategy of hemicyanine-based near-infrared (NIR) fluorophores for conjugation with biochanin A (BCA). The zwitterionic NIR fluorophore (ZW800-Cl) was finally selected from the conventional heptamethine cyanine fluorophores after in silico calculations of their hydrophobicity (log*D* at pH 7.4) values.

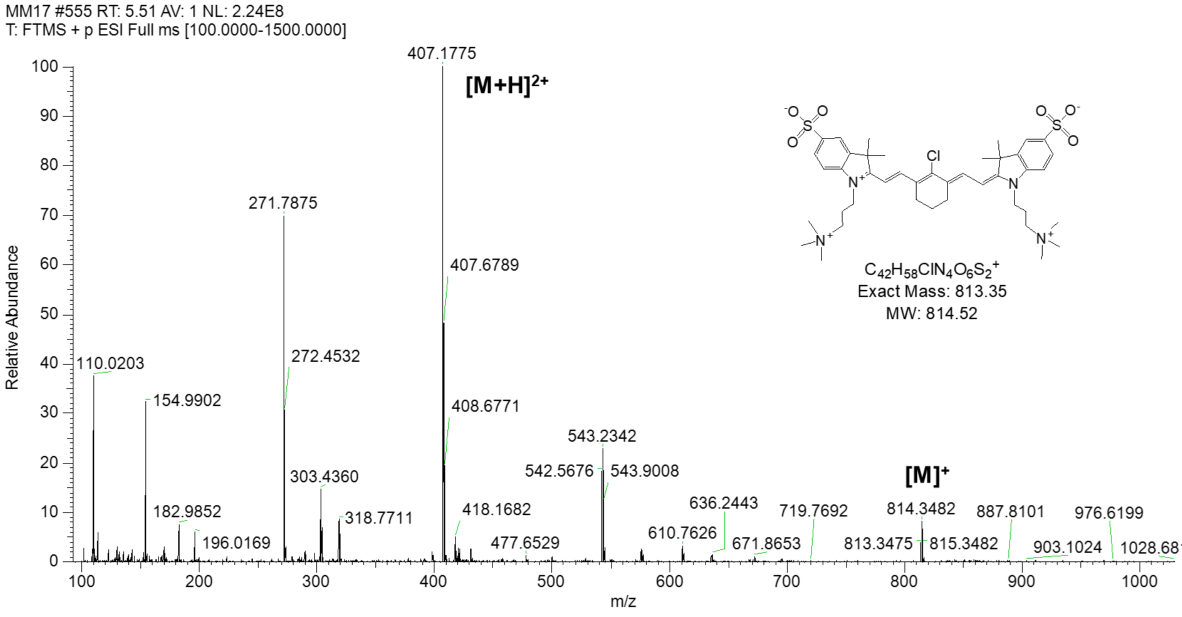

Figure S2. Mass spectrum of ZW800-Cl.

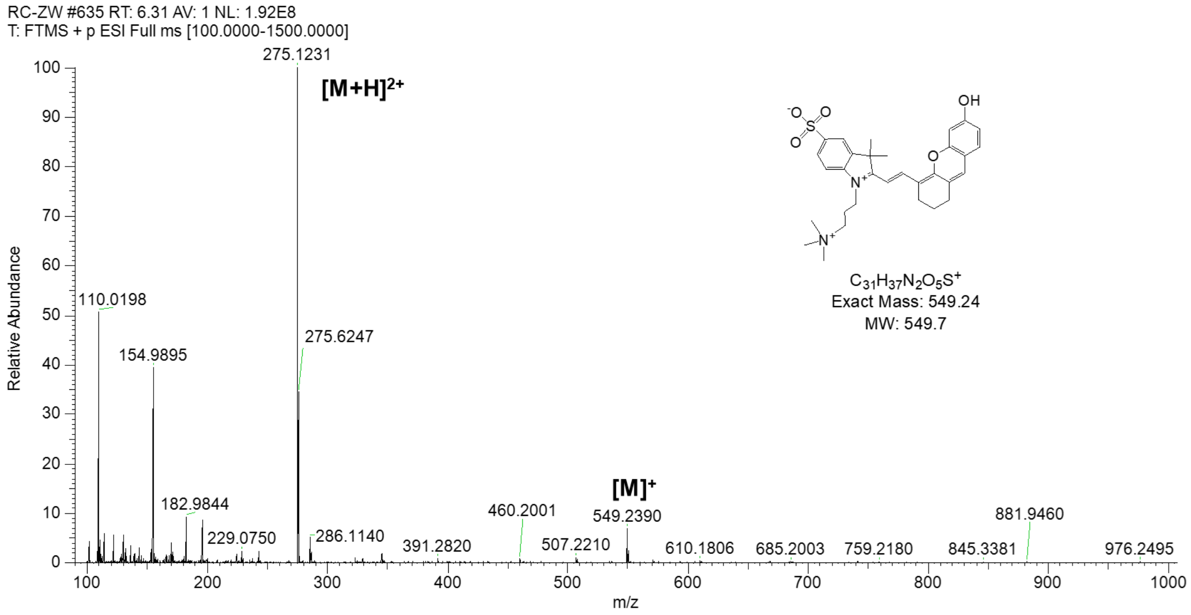

Figure S3. Mass spectrum of RC-ZW.

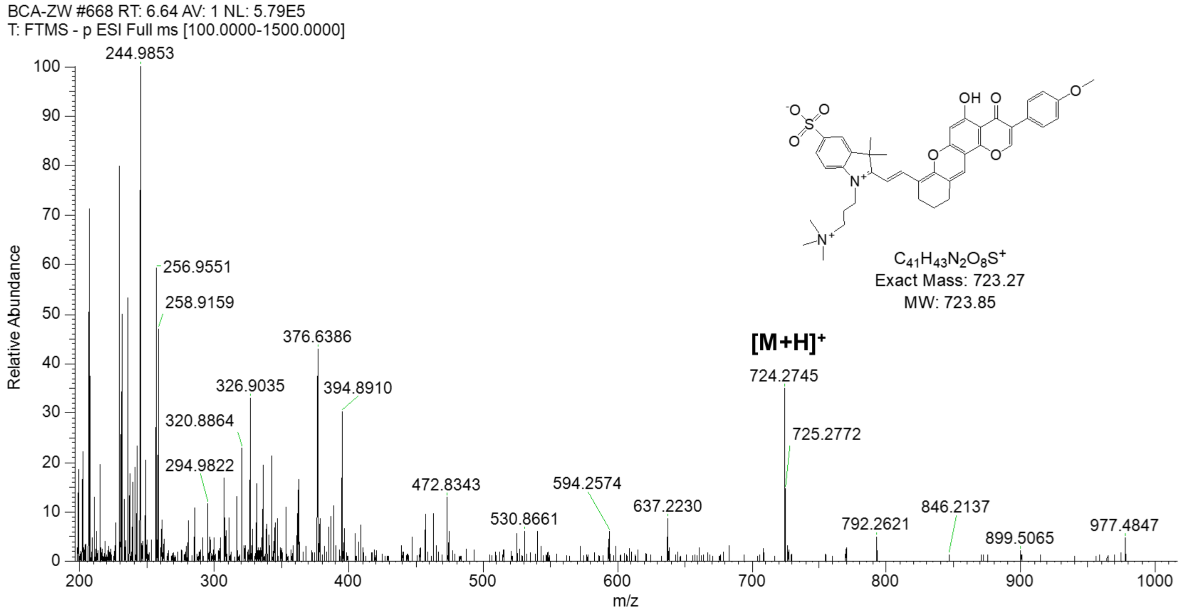

Figure S4. Mass spectrum of BCA-ZW.

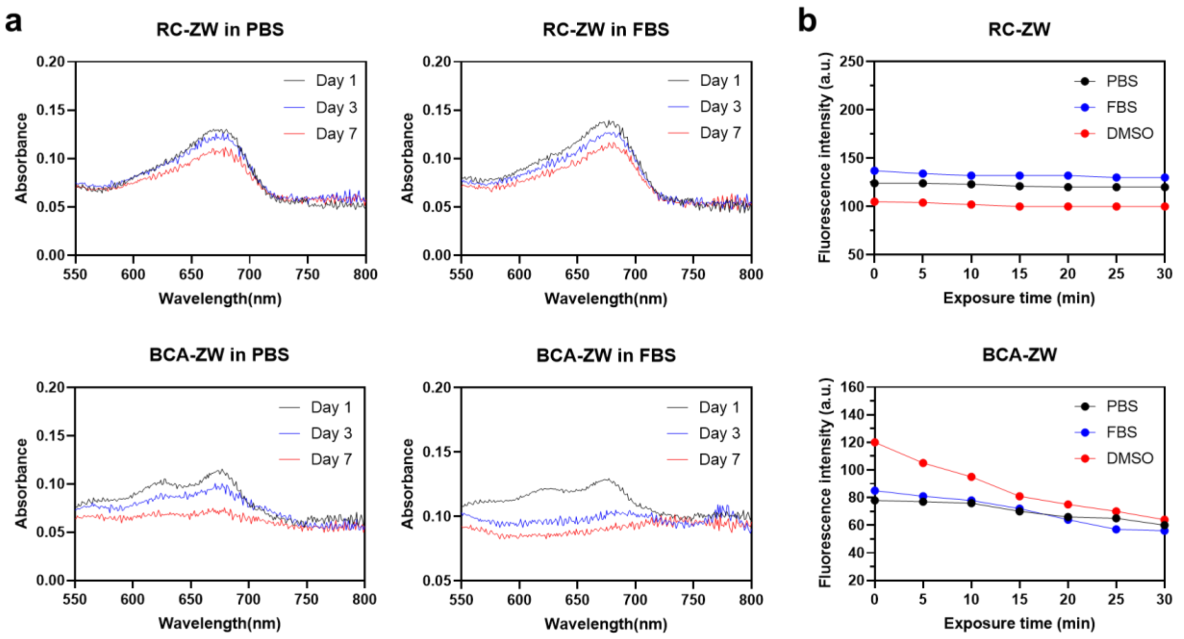

Figure S5. (a) Stability of RC-ZW and BCA-ZW in PBS and FBS. Stability was determined by measuring the absorbance of each sample stored at ambient temperature for 7 days. (b) Photostability of RC-ZW and BCA-ZW in PBS, FBS, and DMSO exposed to NIR light sources in a small animal NIR imaging system (FOBI) for 30 min.

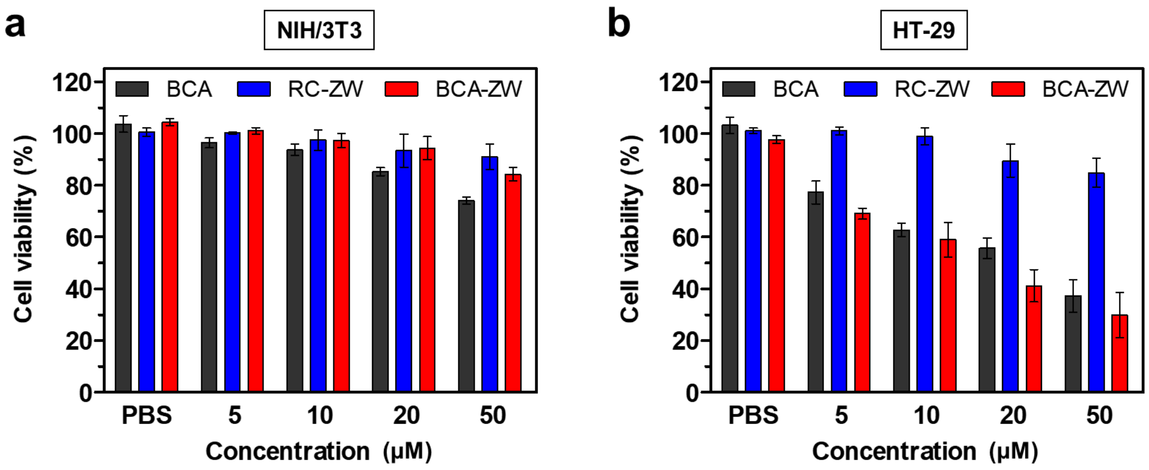

Figure S6. Cell viability assay of BCA, RC-ZW, and BCA-ZW using (a) NIH/3T3 and (b) HT-29 cells, respectively. Percentage cytotoxicity is determined after 24 h of treatment with various concentrations of BCA, RC-ZW, and BCA-ZW.

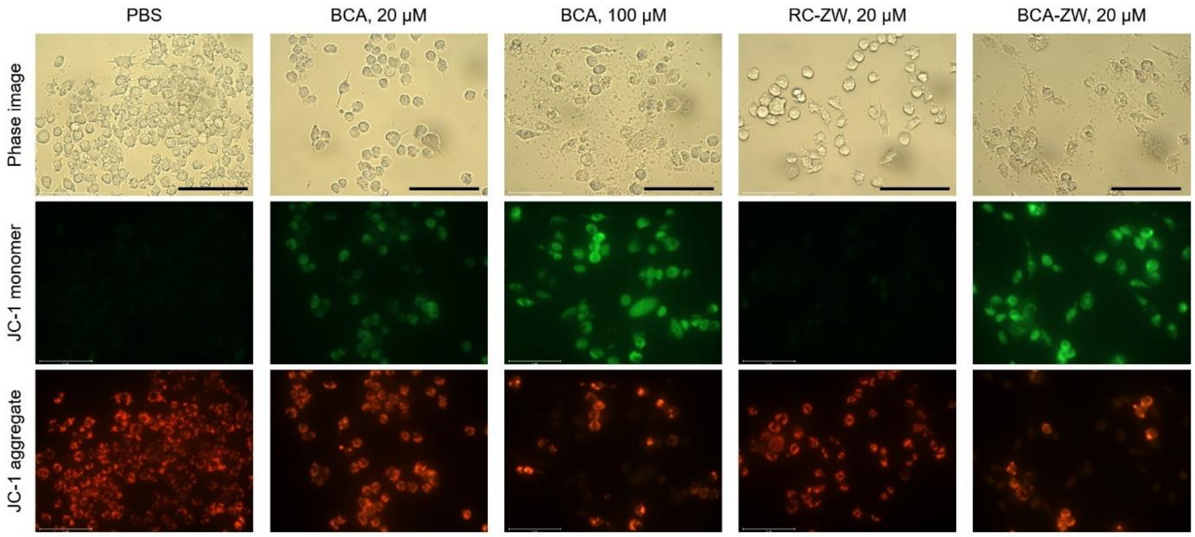

Figure S7. Measurement of mitochondria membrane potential by JC-1 staining. HT-29 cells were incubated with BCA, RC-ZW, and BCA-ZW for 24 h. Scale bars = 100 µm. Images are representative of n = 3 independent experiments. All fluorescence images have identical exposure times and normalization.

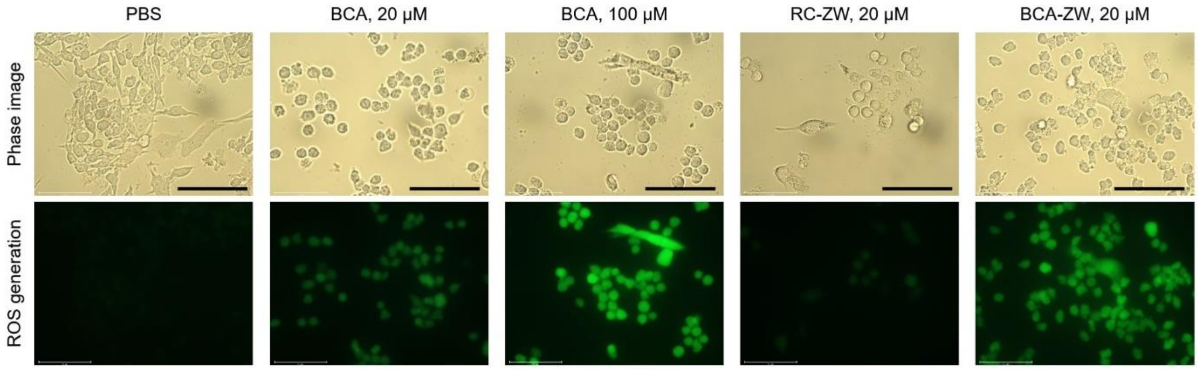

Figure S8. Determination of cellular reactive oxygen species (ROS) by DCF-DA assay. HT-29 cells were incubated with BCA, RC-ZW, and BCA-ZW for 24 h. Scale bars = 100 µm. Images are representative of n = 3 independent experiments. All fluorescence images have identical exposure times and normalization.

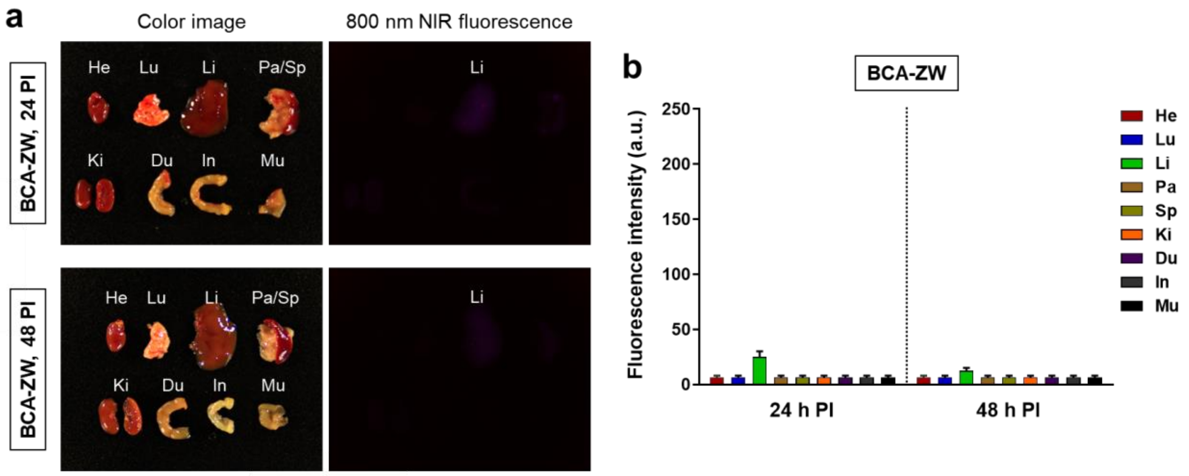

Figure S9. In vivo biodistribution of BCA-ZW. (a) Resected major organs imaged 24 h and 48 h after injection of BCA-ZW, respectively. (b) Quantitative fluorescence analysis of intraoperative dissected organs 24 h and 48 h post-injection of BCA-ZW. Abbreviations: Du, duodenum; He, heart; In, intestine; Ki, kidneys; Li, liver; Lu, lungs; Mu, muscle; Pa, pancreas; Sp, spleen; and PI, post-injection. Images are representative of n = 3 independent experiments. All NIR fluorescence images have identical exposure times and normalization.

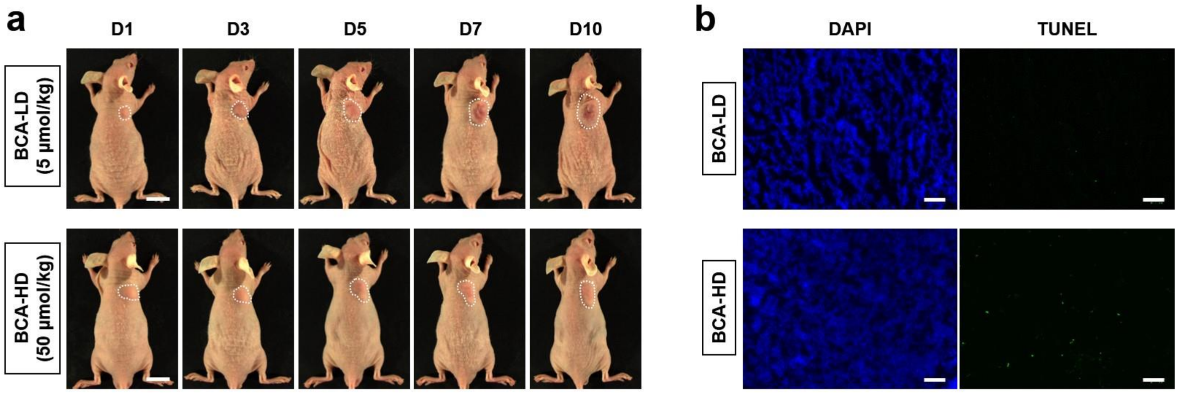

Figure S10. In vivo antitumor efficacy of low-dose (LD; 5 μmol/kg) and high-dose (HD; 50 μmol/kg) BCA. (a) Representative photos of changes in tumor size in HT-29 tumor-bearing mice for 10 days after different treatments. The tumor sizes are indicated by white dotted lines. Scale bars = 1 cm. (b) Apoptosis detection by TUNEL assay in tumor tissues harvested 7 days after different treatments. DAPI staining demonstrated the cell nucleus. Scale bars = 100 μm. Images are representative of n = 3 independent experiments. All fluorescence images have identical exposure times and normalization.

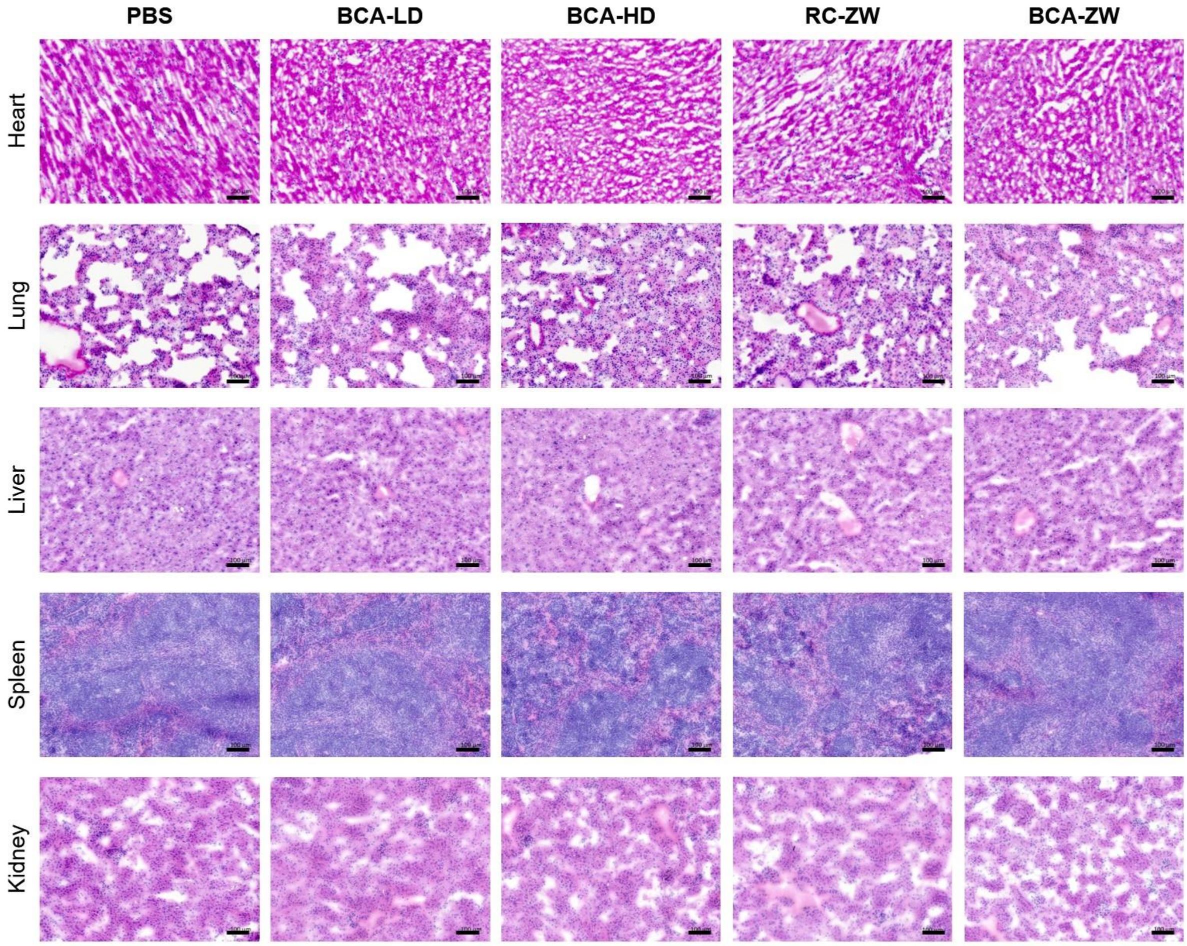

Figure S11. H&E stained images of major organs including heart, lung, liver, spleen, and kidney tissues harvested from each treatment group at day 10. Scale bars = 100 μm. Images are representative of n = 3 independent experiments.

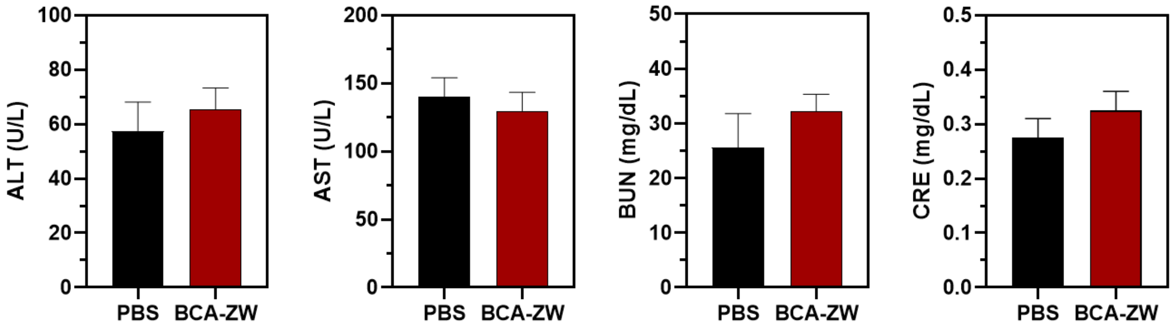

Figure S12. Blood biochemical analysis after intravenous injections of PBS and BCA-ZW.

Supporting References

[S1] H. S. Choi, K. Nasr, S. Alyabyev, D. Feith, J. H. Lee, S. H. Kim, Y. Ashitate, H. Hyun, G. Patonay, L. Strekowski, M. Henary, J. V. Frangioni, *Angew. Chem. Int. Ed.* **2011**, *50*, 6258.

[S2] H. Hyun, M. W. Bordo, K. Nasr, D. Feith, J. H. Lee, S. H. Kim, Y. Ashitate, L. A. Moffitt, M. Rosenberg, M. Henary, H. S. Choi, J. V. Frangioni, *Contrast Media Mol. Imaging* **2012**, *7*, 516.

[S3] J. Huang, Y. Lyu, J. Li, P. Cheng, Y. Jiang, K. Pu, *Angew. Chem. Int. Ed.* **2019**, *58*, 17796.
